# Vitamin A regulates intramuscular adipose tissue and muscle development: promoting high-quality beef production

**DOI:** 10.1186/s40104-021-00558-2

**Published:** 2021-03-05

**Authors:** Dong Qiao Peng, Stephen B. Smith, Hong Gu Lee

**Affiliations:** 1 Department of Animal Science and Technology, Sanghuh College of Life Sciences, Konkuk University, Seoul, 05029 South Korea; 2 Department of Animal Science, Texas A&M University, College Station, TX 77843 USA

**Keywords:** Adipose tissue, Cattle, Intramuscular adipose tissue, Muscle development, Vitamin A

## Abstract

During growth in cattle, the development of intramuscular adipose tissue and muscle is dependent upon cell hyperplasia (increased number of adipocytes) and hypertrophy (increased size of adipocytes). Based on the results of previous studies, other adipose tissue depots (e.g., perirenal and subcutaneous) develop from the fetal stage primarily as brown adipose tissue. The hyperplastic stage of intramuscular adipose is considered to develop from late pregnancy, but there is no evidence indicating that intramuscular adipose tissue develops initially as brown adipose tissue. Hyperplastic growth of intramuscular adipose continues well into postweaning and is dependent on the timing of the transition to grain-based diets; thereafter, the late-stage development of intramuscular adipose tissue is dominated by hypertrophy. For muscle development, hyperplasia of myoblasts lasts from early (following development of somites in the embryo) to middle pregnancy, after which growth of muscle is the result of hypertrophy of myofibers. Vitamin A is a fat-soluble compound that is required for the normal immunologic function, vision, cellular proliferation, and differentiation. Here we review the roles of vitamin A in intramuscular adipose tissue and muscle development in cattle. Vitamin A regulates both hyperplasia and hypertrophy in *in vitro* experiments. Vitamin A supplementation at the early stage and restriction at fattening stage generate opposite effects in the beef cattle. Appropriate vitamin A supplementation and restriction strategy increase intramuscular adipose tissue development (i.e., marbling or intramuscular fat) in some *in vivo* trials. Besides, hyperplasia and hypertrophy of myoblasts/myotubes were affected by vitamin A treatment in *in vitro* trials. Additionally, some studies reported an interaction between the alcohol dehydrogenase-1C *(ADH1C)* genotype and vitamin A feed restriction for the development of marbling and/or intramuscular adipose tissue, which was dependent on the timing and level of vitamin A restriction. Therefore, the feed strategy of vitamin A has the visible impact on the marbling and muscle development in the cattle, which will be helpful to promote the quality of the beef.

## Introduction

In recent years, with the improvement of national economies, more consumers are willing to pay for high-quality beef with satisfaction, and beef cattle producers also benefit from high-quality beef production. In beef production, beef quality and beef quantity are two of the most critical factors in the beef grading system in many countries such as the USA, South Korea, and Japan [1–3]. Vitamin A is a fat-soluble vitamin, which is an essential substance to maintain a healthy vision and primary physiological function of cattle [4, 5]. Vitamin A was first found to be negatively correlated with marbling score in carcasses of Japanese Black steers [6]. This was supported by numerous studies on adipocyte development with vitamin A restriction strategy in beef cattle during fattening period [7–9], as well as research in *in vitro* models [10–12]. In addition, the *in vitro* trial in human and mouse cell model indicated that vitamin A upregulated the preadipocyte genes, but downregulated the adipogenesis level in the [13]. Also, recent study in Angus beef cattle reported that vitamin A supplementation at only birth stage enhanced the final marbling development [14]. These studies suggested that vitamin A feeding strategy might have different effect on marbling development at different stage of the beef cattle. At the same time, the relationship between vitamin A supplementation and muscle development was also investigated in *in vivo* and *in vitro* studies [15, 16]. These studies have demonstrated that vitamin A has a profound impact on adipocyte and muscle development in beef cattle.

## Vitamin A digestion, metabolism, and physiological function

Vitamin A is a fat-soluble compound required in the diet of many animals, including ruminants [5]. Vitamin A can be absorbed by ruminants from plant sources (as carotenoids, or provitamin A) and feed additives (as retinol or retinyl ester, or preformed vitamin A) [17]. In ruminants, after vitamin A compounds enter the small intestine, mixed micelles containing digested lipid components are transported to intestinal mucosal cells [18]. In the enterocytes (mucosal cells), carotenoids and preformed vitamin A are converted to retinol or retinyl ester under the action of multiple enzymes, including β-carotene 15,15′-monooxygenase, lecithin:retinol acyltransferase and acyl-CoA:retinol acyltransferase [19–21]. Retinyl esters are transported to various tissues, including the liver primarily by the lymph circulation system, but also in the vascular system in the form of chylomicrons [22]. In the hepatocytes, retinyl esters contained in chylomicron remnants are taken up and hydrolyzed to retinol for further physiological functions in other target cells [17]. When transported to the target cell, retinol can be reversibly oxidized into retinal by retinol dehydrogenase (RDH) and alcohol dehydrogenase (ADH) [23, 24]. Retinal can be converted to retinoic acid by the oxidation of retinaldehyde by aldehyde dehydrogenases (RALDH or ALDH) [23, 25].

Numerous physiological functions of vitamin A are achieved through retinal and retinoic acid. Retinoic acid regulates gene expression through factors bound to the retinoic acid-responsive element, retinoic acid receptor (RAR), retinoid X receptor (RXR) [24]. All-*trans* and 9-*cis* retinoic acids are the primary isoforms that regulate gene expression [26]. In addition, the cellular retinoic acid-binding proteins (CRABP1 and 2), which can bind retinoic acid efficiently and involve in transporting retinoic acid into the nucleus [27]. Previous paper reviewed that CRABP1 could balance the concentration of the retinoic acid by regulating the catabolism of retinoic acid and mediated the non-transcriptional function of retinoic acid by the CRABP1-retnoic acid complex [28]. CRABP2 might inhibit the cell proliferation by passing retinoic acid to RAR, which also involved in the regulation of the preadipocyte differentiation [28–30]. The excess retinol is stored in stellate cells of the liver after being re-esterified to retinyl esters [26]. The liver provides the primary storage site for vitamin A, and more than 95% of vitamin A exists in the liver in the form of retinyl esters [31]; the stellate cells are responsible for approximately 80% of the total retinyl ester storage [22].

Multiple physiological functions of vitamin A have been determined. Vitamin A participates in the formation of rhodopsin (visual purple) for night vision and is required for normal epithelial cell function [18]. Vitamin A and carotenoids have antioxidant activity via scavenging of peroxyl radicals and singlet oxygen [32, 33]. Other studies reported that retinoids are associated with cell proliferation, differentiation, and gene expression via cytosolic retinol-binding protein and cytosolic retinoic acid-binding proteins [34, 35].

## Fetal and postnatal development of adipose tissue and muscle in bovine calves

### Fetal and postnatal development of brown and white adipose tissue

Brown adipose tissue (BAT) and white adipose tissue (WAT) develop in the second or early third trimester of pregnancy in cattle [36]. Perirenal adipose tissue differentiates initially as BAT, but subcutaneous adipose tissue also expresses uncoupling protein-1 (UCP1, a halmark of BAT) and contains a mixture of brown and white adipocytes [37]. WAT stores energy [33] and BAT primarily is responsible for neonatal heat production [38, 39], and both WAT and BAT are sensitive to maternal nutrition and endocrine environment [36, 40, 41]. An increase in adipose tissue mass involves both hyperplasia (increase in cell number, i.e., adipocyte progenitor cell proliferation) and hypertrophy (increase in cell s ize, i.e., lipogenesis and triglyceride accumulation) [42–44].

There are four primary types of adipose tissue in livestock species: visceral, subcutaneous, intermuscular, and intramuscular adipose tissue (also called marbling) [45]. Visceral adipose tissue (which in this discussion includes perirenal adipose tissue) develops first, followed by subcutaneous, intermuscular, and intramuscular adipose in growing cattle. As indicated above, subcutaneous adipose tissue (and perhaps seam adipose tissue) of newborn calves contain a mixture of brown and white adipocytes, which probably different from the initial fetus period performs as *BAT*. However, fetal muscle does not express *UCP1* [37], so development of fetal muscle is unlikely the intramuscular adipose tissue which develops as *BAT* in the initial fetus period. Progenitor cells for intramuscular adipose tissue are present in bovine muscle, but lipid synthesis and triglyceride accumulation occur postweaning, and not until the calves are transitioned to a high-energy, corn-based diet [46, 47]. Subcutaneous and intramuscular adipose tissue (and probably all adipose tissue depots) retain the capacity to proliferate in mature cattle [48], so bovine adipose tissue mass increases initially by hyperplasia and then mainly by hypertrophy.

### Muscle development during fetal and postnatal stage

In mammals, skeletal muscle represents more than 50% of body mass, and muscle mass is determine by the number and size of the muscle fibers [49]. Skeletal muscle growth is initiated early in embryonic development, at which time myoblasts derived from somites undergo extensive hyperplasia until approximately mid-gestation, followed by increasing myotube and size prenatally [50]. From late gestation onward, muscles increase in mass by hypertrophy as new myofibrils are added to existing muscle fibers [51, 52]. Specifically, the primary embryonic muscle fiber develops from 30 days of gestation and lasted for 3 months, and major myofibers mature into slow type I muscle fibers [53]. Secondary muscle fibers are generated at the termination of primary muscle fiber development and lasts to mid-gestation. These secondary muscle fibers differentiate mostly into fast type II muscle fibers and a few developed into type I fibers, whose fiber type can be converted to other fiber types according to postnatal conditions [54]. The total number of muscle fibers is fixed around the mid-gestation (around 180 days of post-conception in cattle) [54, 55]. Tertiary muscle fiber development starts from 110 days of gestation and maintains an undifferentiated state at birth, based on the fast and slow myosin heavy chain (*MyHC*) isoform expression of fibers in the bovine muscle [49, 54, 55]. It was reported that cattle had type I, IIa, and IIX types of mature *MyHC* isoforms 3 weeks after birth [49, 54, 55].

## Vitamin A and intramuscular adipose tissue hyperplasia and hypertrophy

Marbling (also referred to intramuscular fat) is agglomerated white streaks and flecks across the muscle surface [56], which profoundly affects the juiciness, tenderness, and flavor of the beef [57, 58]. The accumulation of marbling is dependent on an increase in intramuscular adipocyte development, which frequently is associated with an increase in monounsaturated fatty acids [59].

### Vitamin A supplementation and intramuscular adipose tissue development in cattle

The relationship between vitamin A and marbling score was firstly observed in Japanese Black cattle; vitamin A restriction during the fattening period increased marbling development [6]. Subsequently, other studies addressing the effects of vitamin A status and marbling score were conducted in other breed types of cattle, such as Angus crossbred steers, Simmental steers, feedlot heifers [7, 8], and Korean native steers [9] (Table 1). Some studies indicated an increase in marbling with vitamin A restriction, but other studies reported no significant effects with vitamin A restriction. There was a fact ignored for many years that pro-vitamin A (carotenoids) in feedstuffs might contain a large amount of vitamin A equivalents which could limit the effect of vitamin A restriction. The extensive assessment of vitamin A content in the feedstuff need to be conducted for a better vitamin A restriction in the beef cattle [65]. It appears that a very low level or no vitamin A supplementation (compared to National Research Council (NRC) requirements for beef cattle), including low or no supplementation before vitamin A treatments, were necessary during the vitamin A restriction period to elevate marbling scores and/or intramuscular fat (IMF) during the fattening period [9, 60, 65–67].

**Table 1:** Studies reporting vitamin A restriction and marbling or intramuscular fat (IMF) in cattle

Breed type	Treatment	General results/conclusions	Reference
Japanese Black steers	Backgrounded at about 20,000 IU vitamin A/day, low vitamin A (no extra supplementation) diet was fed from 15 to 23 months of age	Maintaining a low level of serum vitamin A (≤ 20 μg/dL) before 23 months of age may contribute to higher marbling scores	[6]
Black yearling steers	Backgrounded at 1364 IU vitamin A/kg DM, then 0, 1103, 2205, 4410, or 8820 IU supplemental vitamin/kg DM was fed for 142 or 143 days	Little effect was observed for performance, marbling, or lipogenic enzyme activity in adipose tissue	[8]
Korean native steers	Backgrounded at 8890 IU vitamin A/kg DM, then 8890 (Con), 3890 (T1), or 890 (T2) IU restricted vitamin A/kg DM was fed for 240 days	Marbling scores in the vitamin A-restricted groups were not significantly higher than in the control group but showed a trend for a higher yield grade	[9]
Feedlot heifers	Backgrounded at 3640 IU vitamin A/kg DM, then 3640 or 0 IU vitamin A/kg DM was fed for 218 days	Vitamin A restriction increased the degree of marbling without affecting backfat thickness	[60]
Holstein steers	Backgrounded at 2700 IU vitamin A/kg DM, then 2200 or 950 IU of vitamin A/kg DM was fed for 112 days or 243 days (2200 IU for 112 days and 950 IU for latter 131 days)	Feeding the restricted vitamin A 243 days increased IMF without affecting subcutaneous or visceral fat deposition	[61]
Feedlot heifers	Backgrounded at < 1300 IU vitamin A/kg DM, then 0 or 2700 IU supplemental vitamin A/kg DM was fed for 168 days	The percentage of carcasses grading ≥ Choice^−^was not different between treatment groups	[62]
Feedlot heifers	Backgrounded at < 1100 IU vitamin A/kg DM, then 0 or 2700 IU supplemental vitamin A/kg DM was fed for 112 or 227 days	Vitamin A restriction did not affect USDA yield grade or marbling score	[63]
Angus steers	Backgrounded at a low β-carotene and vitamin A cereal-based ration (no detailed data), then 0 or 60,000 IU supplemental vitamin A/100 kg of BW/day was fed for 308 days	There was greater IMF in longissimus thoracis et lumborum and greater seam fat, but there ws no significant change in marbling score	[64]

A review of the nutrigenomic regulation of adipose tissue hypothesized that marbling development was initiated during the prenatal stage (late pregnancy) and postnatal stage (until about 250 days), which was associated with preadipocyte hyperplasia [68]. Oral administration of vitamin A (78,000 IU/d) during late-stage pregnancy increased birth weight of calves and increased the mRNA expression related to muscle [myogenic factor 5 (*Myf5*)*,* myogenic factor 6 (*Myf6*)*,* myoblast determination protein 1 (*MyoD*)] and preadipocyte (Krüppel like factor 2 (*KLF2*)] development [69]. Vitamin A injection at birth and one-month age in Angus calves resulted in elevated intramuscular adipose tissue deposition in the carcass trait result when averagely slaughtered at day 438 [14, 70]. Our previous study also reported that oral vitamin A supplementation also enhanced the growth performance of the neonatal Korean native calves, as well as promoted the preadipocyte [zinc finger protein 423 (*Zfp423),* preadipocyte factor 1 *(Pref-1)*] and muscle (*MyoD, Myf6, myogenin*) development in the genetic level [71]. Besides, vitamin A intake in the early growth stage in rats led to greater adiposity in rats [70]. These results implied that vitamin A supplementation in the late pregnancy and neonatal calves enhances the preadipocyte development, which further indirectly promotes the intramuscular adipose tissue deposition in the late stage of cattle. Therefore, these studies indicate that during early developmental stage, vitamin A supplementation promotes the adipogenic progenitor cells by preadipocyte hyperplasia process. However, in the fattening stage, vitamin A supplementation stimulates the lipid oxidation, thus restriction of vitamin A increases the lipid accumulation by the adipocyte hypertrophy.

### Vitamin A, preadipocyte hyperplasia, and adipocyte hypertrophy

**Fig. 1:**
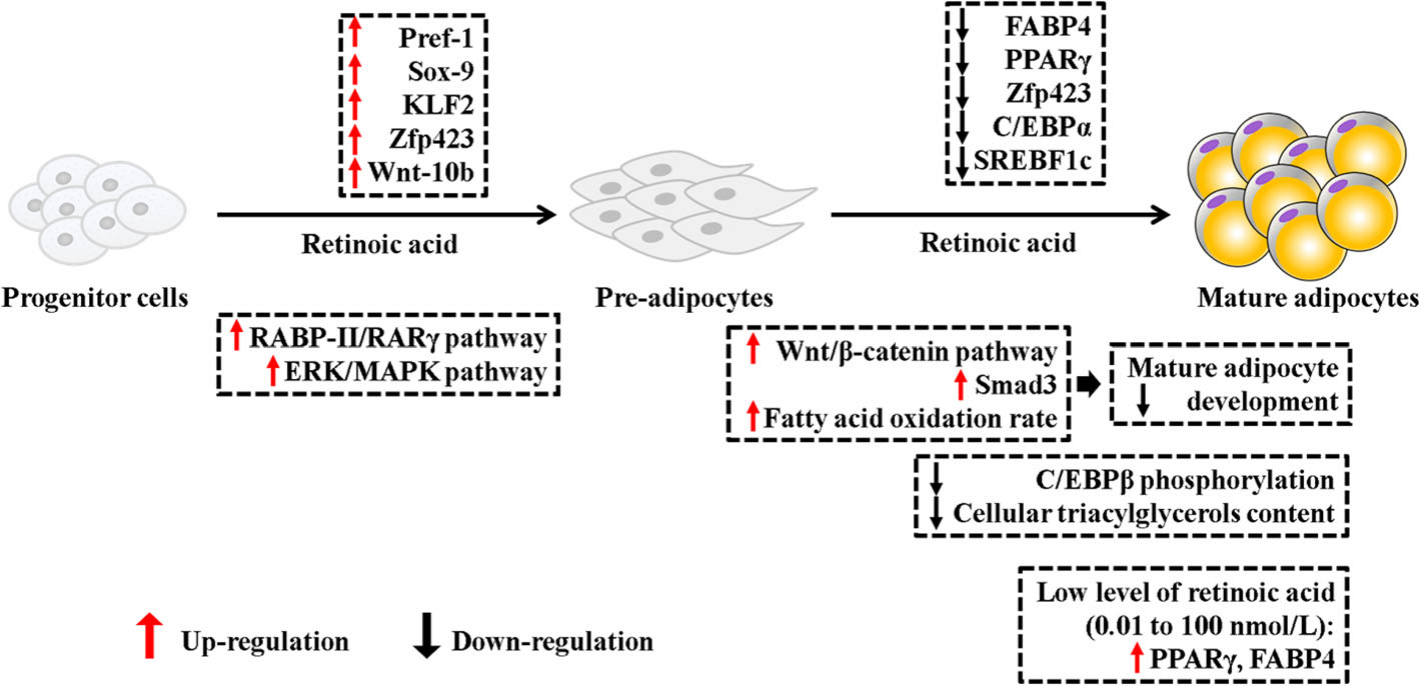
Retinoic acid supplementation plays various roles that vary from preadipocyte development to the adipocyte deposition. The full names of genes are preadipocyte factor-1 (Pref-1), Krüppel-like factor 2 (KLF2), zinc finger protein 423 (Zfp423), Wnt family member 10b (Wnt-10b), fatty acid binding protein 4 (FABP4), peroxisome proliferator-activated receptor gamma (PPARγ), CCAAT/enhancer-binding protein alpha/beta (C/EBPα/β), sterol regulatory element binding protein 1c (SREBF1c), extracellular signal-regulated kinase 1/2 (ERK), mitogen-activated protein kinase (MAPK), retinoic acid binding protein (RABP), retinoic acid receptor gamma (RARγ)

There have been several investigations of the effects of vitamin A on adipogenesis and subsequent development of intramuscular adipose tissue, which are summarized in Fig. 1. During the adipogenic differentiation period (adipocyte hypertrophy), the effects of retinoic acid are regulated by the *RAR* and *RXR* families in the promoter region of target genes [72, 73]. In particular, all-*trans*-retinoic acid supplementation inhibited the expression of adipogenic marker genes, such as fatty acid binding protein 4 *(FABP4)*, peroxisome proliferator-activated receptor gamma* (PPARγ)*, and CCAAT/enhancer-binding protein alpha *(C/EBPα)* during the early differentiation stage [74]. The retinoic acid inhibits lipid accumulation by stimulating PPAR*α/β/δ i*n mature adipocytes which relates to lipid oxidation and catabolism [68]. As well as activating the Wnt/β-catenin signaling pathway [Wnt family member 1 *(Wnt-1),* Wnt family member 4 *(Wnt-4)*, Wnt family member 10 *(Wnt10b)*, and β-catenin] in 3T3-L1 cells, which may associate with the adipogenesis suppression induced by retinoic acid [11]. Supplementation of 9*-cis* retinoic acid inhibits adipogenesis by decreasing the *PPARγ* and *RXR* levels in 3 T3-L1 cells [74] and induced liver *FABP* gene expression above the induction caused by oleic acid in the subconfluent rat hepatoma cells [75]. In addition, all*-trans* retinoic acid supplementation activated *RAR *and peroxisome proliferator-activated receptor beta/delta *(PPARβ/δ)* in mature adipocytes [76], stimulated transforming growth factor β-effector protein (mothers against decapentaplegic homolog 3*, Smad3*), blocked the phosphorylation of CCAAT/enhancer-binding protein beta *(C/EBPβ)*, thereby decreasing the adipogenesis [77, 78]. Vitamin A reduced cellular triacylglycerol content and upregulated the fatty acid oxidation rate by increasing the expression of *UCP* gene family [10, 79]. Also, demethylation in the promotor of *Zfp423* was disrupted by retinoic acid [80]. Interestingly, a recent study demonstrated that retinoic acid supplementation regulates adipogenesis and cell proliferation through both positive and negative functions, which depended on retinoic acid dosage during the differentiation period [81]; fatty acid oxidation only occurred at specific concentrations.

More attention has been paid to the influence of retinoic acid on preadipocyte hyperplasia in recent years. Previous studies demonstrated that *Pref-1*, *Sox-9*, *Wnt-10b*, *Zfp423*, and *KLF2* were expressed at high levels in preadipocytes but not in mature adipocytes [82–84]. Retinoic acid inhibited adipocyte differentiation by activation of the cellular retinoic acid-binding protein 2/retinoic acid receptor *(CRABP-II/RAR)* pathway, but decreased the expression of *Pref-1*, *Sox-9*, and *KLF2* in mature adipocytes, which typically are highly expressed only in preadipocytes [13]. Also, the activation of the *ERK/MAPK* pathway was required during the adipogenic commitment process by retinoic acid treatment in embryonic stem cells [85]. These results were summarized in Fig. 1, which shows that retinoic acid plays a crucial role in the development of the preadipocyte hyperplasia by activating related genes and stimulating the progenitor proliferation by the signaling pathway. When come to the adipogenic differentiation period, retinoic acid shows the inhibition of lipid accumulation and down-regulated the related genes expression. Paradoxically, low levels of vitamin A supplementation also promotes adipogenesis.

## Vitamin A, myoblast hyperplasia, and myotube differentiation

Taylor and Jones [86] demonstrated that myoblasts and adipocytes can be derived from the same mesodermal stem cell precursors, which is dependent on upregulation of *Wnt* signalling [87]. Mesodermal stem cell precursors are activated by paired box (*Pax*) 3 and 7 during somitogenesis [88]. The muscle lineage is determined by the muscle-specific transcription *MyoD* and *Myf5* at an early stage of embryonic development [89]. Followed by proliferation, a pool of myoblasts is formed following the expression of *Myf5* and *MyoD* genes in the initial stage of muscle differentiation [90]. Following terminal differentiation, multinucleated myotubes are established by the expression of muscle-specific genes such as *Myf6*, myogenin, and myocyte enhancer factor 2 *(MEF2)* genes [91]. Subsequently, myotubes form myofibers following extensive myobril synthesis [92, 93].

Metabolites of retinoic acid can promote myogenic differentiation [88, 94]. In zebrafish, the retinoic acid activates muscle differentiation *in vivo* through fibroblast growth factor 8 *(Fgf8)* gene signaling, a muscle differentiation activator; suppression of retinoic acid signaling inhibits muscle differentiation and *myoD* expression [95]. The ovine primary myoblasts, retinoic acid dramatically decreased cell proliferation by a reduction of cyclin D1 protein, but retinoic acid increased myogenin gene expression and MyHC protein levels, and upregulated the glucose transporter 4 (*GLUT4*) mRNA and protein expression [96]. Retinoic acid treatment of human embryonic stem cells expanded the premyogenic progenitor population, promoting the *Pax3*-positive myoblast population and elevating the expression of *MyoD*, myogenin, and mesenchyme homeobox 1 *(Meox1)* [97]. In addition, a high dose level of retinoic acid supplementation in the C2C12 cell line still stimulated the myogenic differentiation, but the MyHC protein level was obviously decreased [98].

All-*trans*-retinoic acid promoted myogenin expression but had no effect on the cell cycle arrest in the rhabdomyosarcoma cell line [99, 100]. Maternal vitamin A deficiency in rats did not affect fetal weight but decreased survival rate as well as downregulated the protein level of Myf5, myogenin, and MyHC expression compared to rats receiving moderate vitamin A supplementation [101]. In addition, previous study in Black Angus steer calves reported that vitamin A injection to the neonatal calves upregulated the expression of myogenic genes (*Pax3*, *Pax7*, *Myf5*, *MyoD* and myogenin) [15]. Besides, our previous study also showed that oral vitamin A supplementation to the Korean native calves enhanced the expression of *MyoD*, *Myf6* and myogenin in the *longissimus dorsi* muscle as well as the growth performance [71].

**Fig. 2:**
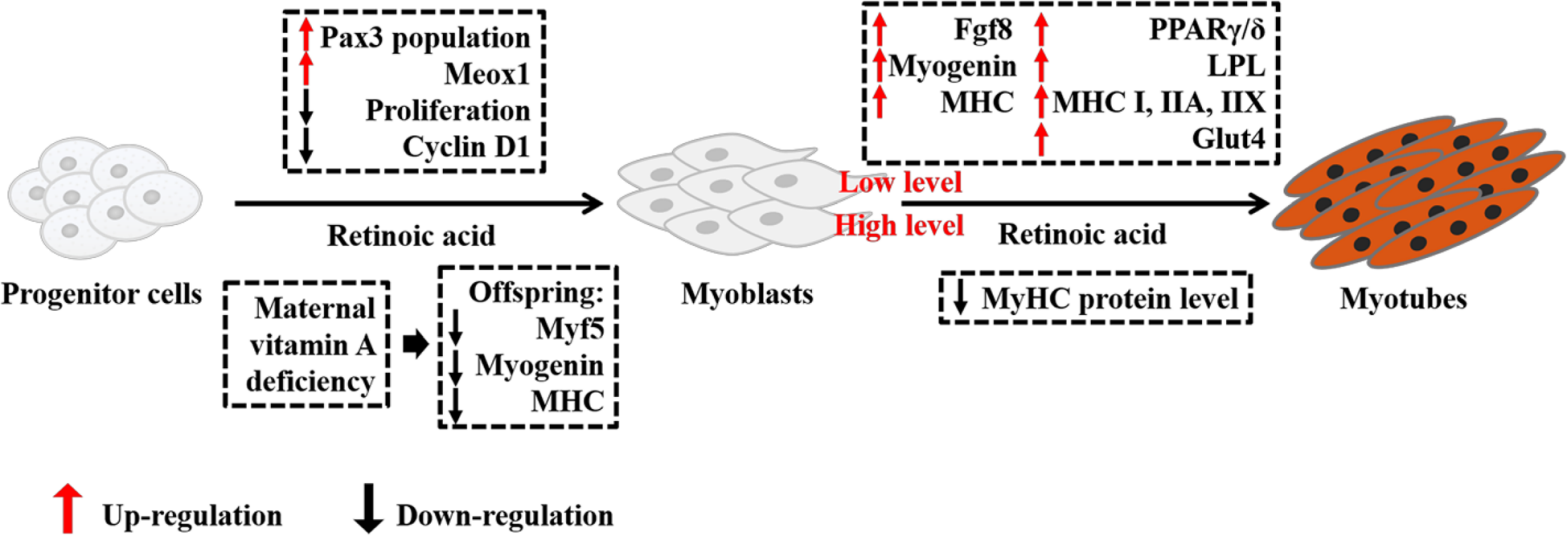
Retinoic acid supplementation plays various roles that vary from myoblasts development to myogenesis stage. The full names of genes are paired box gene 3 (Pax3), mesenchyme homeobox 1 (Meox1), fibroblast growth factor 8 (Fgf8), major histocompatibility complex (MHC), peroxisome proliferator-activated receptor gamma/delta (PPARγ/δ), lipoprotein lipase (LPL), glucose transporter type 4 (Glut 4), myogenic factor 5 (Myf5), myoblast determination protein (MyoD), Wnt family member 5a (Wnt5a), Ca^2+^/calmodulin-dependent protein kinase II (CaMK2)

In Fig. 2, we conclude that retinoic acid plays an essential role myogenic commitment from the progenitor cells, the deficiency of retinoic acid may decrease the later myogenesis in the genetic levels. In terminal myogenesis, low levels of vitamin A promotes the formation of the myotubes as well as the related gene expression, however high levels of supplementation may have the side effect on maturation of myotubes by influencing the MyHC protein level.

## Vitamin A and metabolites in cattle

Besides, with the deepening research of marbling in cattle, various potential biochemical parameters have been found related to the marbling score under vitamin A treatment. Adachi et al. [102] reported that with in the case of vitamin A restriction, the high serum concentration of glucose, urea nitrogen, albumin/globulin ratio and magnesium showed in the high marbling group than that in control group. Another monitor research in cattle by our lab revealed that, vitamin A restriction led the increase of the level of calcium, total cholesterol, albumin, blood urea nitrogen, creatinine and non-esterified fatty acids in serum [9, 103]. These discovered metabolic indicators may contribute to the marbling production in cattle as well as the metabolic imbalances during the vitamin A restriction. Recently, the other latest research in our lab indicated that vitamin A supplementation in the early growth period of calf increased the levels of cholesterol and myo-inositol both in serum and longissimus dorsi muscle to maintain the preadipocyte status [104].

## Vitamin A, marbling, and the ADH1C genotype in cattle

As stated above, retinol is oxidized into retinaldehyde by RDH and ADH, and retinaldehyde is further oxidized into retinoic acid by RALDH and ALDH [17]. Serum retinaldehyde concentration was dramatically lower in the *RALDH1*^*−/−*^*/ADH1*^*−/−*^ mouse than in wild-type mice [105]. An *ADH1C* SNP was reported in Angus steers, including TT, TC, and CC genotypes. The TT genotype was more common than the CC genotype, with the TC genotype intermediate [106, 107]. The result suggested that the lower performance of the C allele might reduce the transcription of the *ADH1C* gene by elimating a potential binding site of *C/EBPα* [108]. Under vitamin A restriction, the TT genotype of *ADH1C* resulted in a higher level of intramuscular fat than the CC and CT genotypes [106]. A subsequent study in Angus steers reported that 75% of NRC recommended vitamin A supplementation caused greater intramuscular fat for Angus crossbred steers with the *ADH1C* TT genotype of *ADH1C* gene than steers with the CT or CC genotype [106]. However, vitamin A restriction at 25% or 50% of NRC recommendations had no effect on final levels of intramuscular fat [107].

All-*trans*-retinoic acid increases the expression of *PPARγ/δ*, *LPL*, *SMAD3*, and *MyHCIIX* in cultured bovine satellite cells at relatively high doses, and increases *MyHCI* and *MyHCIIA* expression at low levels via the PPARδ pathway [16]. High marbling scores in Korean native cattle (Hanwoo) are associated with a greater percentage of type I muscle fibers and fewer type IIB muscle fibers [109], which may be related to the level of retinoic acid supplementation during production. In a study in Korean native steers, steers with the *ADH1C* TC genotype had higher marbling score than the TT genotype under vitamin A restriction (930 IU/kg of DM); the *ADH1C* CC genotype was not detected in this group of Korean native cattle [110].

Table 2 summarizes the results from the only four published reports of the interaction between ADH1C phenotype and vitamin A supplementation. Three studies [106, 107, 110] observed an increase in marbling with vitamin A restriction, but the *ADH1C* genotype that was affected by vitamin A restriction (TT or TC) differed among these studies. One large study from Canada [111] demonstrated no effect of vitamin A restriction (to 50% recommended levels); nor did observe a vitamin A × *ADH1C* (TT, TC, and CC genotypes) interaction for marbling. Breed types, basal diets, and countries in which the experiments were conducted may have led to the differing results. Moreover, these inconsistencies probably caused by the different effects of retinoic acid in preadipocyte hyperplasia and hypertrophy among these different vitamin A treated levels and different experimental periods. In spite of the inconsistent results among studies, subsequent experiments are warranted to study the interesting interaction between *ADH1C* genotypes and dietary vitamin A levels.

**Table 2:** Studies reporting vitamin A restriction and the ADH1C genotype in cattle

Experimental details	Treatments	Effects on marbling or intramuscular fat (IMF)	Reference
Angus-cross steers (*n* = 130; 50 TT, 50 TC, 30 CC)	Backgrounded at 549 IU vitamin A/kg DM then fed 0 or 2200 supplemental IU vitamin A/kg DM	Un-supplemented steers had greater marbling scores and IMF than supplemented steers. There was a significant treatment × genotype interaction for IMF. With no vitamin A supplementation, TT steers had 23% greater IMF than CC steers. Un-supplemented TT steers had 24% greater IMF than supplemented TT steers	[106]
Angus-cross steers (*n* = 117; 45 TT, 45 TC, 27 CC)	Backgrounded at 3360 IU vitamin A/kg DM, then fed 550, 1100, or 1650 total IU vitamin A/kg DM	A treatment × genotype interaction was observed for IMF; TT steers on the 1650 IU/kg DM treatment had higher IMF relative to CT and CC steers on the same treatment	[107]
Korean native steers (*n* = 136; 102 TT, 34 TC)	Backgrounded at 890 IU vitamin A/kg DM, then fed 930 IU total vitamin A/kg DM	Marbling scores were greater for the TC genotype than the TT genotype following a vitamin A-restricted diet	[110]
Black Angus steers (*n* = 2000; TT and TC), mixed breed	Backgrounded on 3360 IU vitamin A/kg DM, then fed 1100 or 2200 IU total vitamin A/kg DM	There was not a significant vitamin A × ADH1C interaction for marbling score	[111]

## Limitations and conclusion

According to the previous studies, vitamin A feeding strategy played a crucial role on the development of marbling or intramuscular fat in cattle. Several *in vivo* studies documented that vitamin A restriction of cattle during the fattening period increased intramuscular fat in carcasses (by adipocyte hypertrophy), but the broad applicability of vitamin A restriction in beef cattle production is equivocal and warrants further investigation. Current study suggested that vitamin A restriction did not affect backfat depth or lean mass. Furthermore, the effect of vitamin A on preadipocyte hyperplasia was reported in limited studies in beef cattle, however, the inside mechanism by *in vivo* or *in vitro* trials remains to be determined. In addition, existing data suggests that vitamin A contributes to myogenic commitment from progenitor cells and myogenesis in a narrow level of vitamin A supplementation. Although some studies in mouse and fish demonstrated that vitamin A would increase muscle development, few experiments have shown that vitamin A is efficacious for promoting muscle growth in cattle. These findings suggest that vitamin A supplementation during the early stage enhances the preadipocyte hyperplasia, but the restriction of vitamin A during the fattening period increases the lipid accumulation by adipocyte hypertrophy. A limited number of studies demonstrated that *ADH1C* genotype (TT and TC, depending on breed type) contributes to marbling production under vitamin A restriction, and again results are equivocal. Larger studies comparing the effects of vitamin A restriction between different breed types (e.g., Korean native cattle and Holstein or Angus cattle) should be encouraged.

## Data Availability

Not applicable.

## Abbreviations

ADH1C: Alcohol dehydrogenase-1C
RDH: Retinol dehydrogenase
ADH: Alcohol dehydrogenase
RALDH: Retinaldehyde
ALDH: Aldehyde dehydrogenases
RAR: Retinoic acid receptor
RARγ: Retinoic acid receptor gamma
RXR: Retinoid X receptor
CRABP: Cellular retinoic acid-binding proteins (1 and 2)
BAT: Brown adipose tissue
UCP1: Uncoupling protein-1
WAT: White adipose tissue
MyHC: Myosin heavy chain
NRC: National Research Council
IMF: Intramuscular fat
Myf5/6: Myogenic factor 5/6
MyoD: Myoblast determination protein 1
KLF2: Krüppel-like factor 2
Zfp423: Zinc finger protein 423
Pref-1: Preadipocyte factor-1
FABP4: Fatty acid binding protein 4
PPARα/β/γ/δ: Peroxisome proliferator-activated receptor alpha/beta/gamma/delta
C/EBPα/β: CCAAT/enhancer-binding protein alpha/beta
Wnt-1/4/10b: Wnt family member 1/4/10b
Smad3: Mothers against decapentaplegic homolog 3
SREBF1c: Sterol regulatory element binding protein 1c
ERK 1/2: Extracellular signal-regulated kinase 1/2
MAPK: Mitogen-activated protein kinase
RABP: Retinoic acid binding protein
Pax3/7: Paired box gene 3/7
Meox1: Mesenchyme homeobox 1
Fgf8: Fibroblast growth factor 8
MHC: Major histocompatibility complex
LPL: Lipoprotein lipase
GLUT4: Glucose transporter type 4
MEF2: Myocyte enhancer factor 2
